# Medical encounters for opioid-related intoxications in Southern Nevada: sociodemographic and clinical correlates

**DOI:** 10.1186/s12913-016-1692-z

**Published:** 2016-08-24

**Authors:** Jing Feng, Joseph P. Iser, Wei Yang

**Affiliations:** 1 Southern Nevada Health District, PO Box 3902, Las Vegas, NV 89032 USA; 2 School of Community Health Sciences/MS274, University of Nevada, Reno, Reno, NV 89557 USA

**Keywords:** Opioid, Intoxication, Medical encounter, Comorbidity

## Abstract

**Background:**

Despite today’s heightened concern over opioid overdose, the lack of population-based data examining clinical and contextual factors associated with opioid use represents a knowledge gap with relevance to prevention and treatment interventions. We sought to quantify rates of emergency department (ED) visits and inpatient hospitalizations for harmful opioid effects and their sociodemographic differentials as well as clinical correlates in Southern Nevada, using ED visit and hospital inpatient discharge records from 2011 to 2013.

**Methods:**

Cases were identified by ICD-9-CM diagnosis codes for opioid poisoning and opioid-type drug dependence and abuse as well as poisoning and adverse effect E-codes. Comorbid conditions, including pain-related diagnoses, major chronic diseases, affective disorders, sleep disorders, sexually transmitted infections and viral hepatitis were assessed from all available diagnosis fields. Counts by age-race per zip code were modeled by negative binomial regression. Opioid injuries were further examined as a function both of neighborhood income and individual characteristics, with mixed-effects logistic regression to estimate the likelihood for an adverse outcome.

**Results:**

Opioid intoxications and comorbidities were more common in low-income communities. The multivariable-adjusted rate for opioid-related healthcare utilization was 42 % higher in the poorest vs. richest quartile during the study period. The inter-quartile (quartile 1 vs. 4) rate increases for chronic bodily pains (44 %), hypertension (89 %), renal failure/diabetes (2.6 times), chronic lower respiratory disease (2.2 times), and affective disorders (57 %) were statistically significant. Chronic disease comorbidity was greater among non-Hispanic blacks, whereas abuse/dependence related disorders, alcohol or benzodiazepine co-use, chronic bodily pains, and affective disorders were more prevalent among non-Hispanic whites than nonwhites.

**Conclusions:**

There were consistent patterns of disparities in healthcare utilization across sociodemographic groups for opioid-associated disorders. Further initiatives to evaluate the determinants of overdose and abuse and to implement targeted response efforts are needed.

## Background

Drug-related poisonings have been recognized as a veritable epidemic in Clark County (coextensive with the Las Vegas–Paradise Metropolitan Statistical Area [MSA]), Southern Nevada, one of the most populous counties in the United States (US). Since 2005, fatal drug poisonings have surpassed firearm injuries as the leading cause of injury deaths, and mortality from drug overdoses and opioid poisonings in the county were 50 and 70 % higher than comparable national rates in 2012–2014, respectively [1]. Further, the excess mortality from drug-related poisonings in Clark County has been linked to a higher opioid prescribing rate in NV when compared with prescribing patterns nationwide [2]. In view of these alarming statistics, the Nevada legislature enacted an opioid overdose prevention policy in May, 2015, strengthening prescription drug monitoring programs use and physician education programs, and expanding access to naloxone, an opioid antagonist for reversing opioid overdose in out-of-hospital settings.

Despite today’s heightened concern over opioid overdose, the lack of population-based data examining clinical and contextual factors associated with opioid use represents a knowledge gap with relevance to prevention and treatment interventions. While risk factors and trends for opioid-related fatalities have been reported in limited populations [3, 4], fatal poisonings represent only a small fraction of the potential adverse outcomes associated with opioid use. Several studies have examined the incidence and characteristics of hospitalizations and emergency department (ED) visits for opioid overdose [5–7]; however, epidemiologic data on correlates and comorbidities of opioid intoxication remain limited.

The purpose of this study is to examine medical encounters due to harmful opioid effects among major sociodemographic subgroups in Southern Nevada, with a focus on disorders comorbid with opioid use. Medical encounter data can be used to improve our understanding of the epidemiology of opioid overdose, and help delineate groups at risk of adverse outcomes for targeted interventions. Such interventions are urgently warranted as a component in a comprehensive response to this public health crisis.

## Methods

### Data

De-identified inpatient hospitalizations (IH) and ED visit records of 2011–2013 were abstracted from the statewide inpatient and ED databases for Clark County residents. The inpatient and ED databases contain data for approximately 200,000 IHs and 500,000 ED visits in Clark County each year. Approximately 13 and 20 % of the principal (first-listed) diagnoses for IHs and ED visits are for some type of injury, with 87 and 91 % of these injury-attributable inpatient or ED discharges having an external cause of injury code (E-code), respectively.

### Study endpoints

Hospitalizations and ED visits for opioid intoxication were identified by ICD-9-CM diagnosis codes for opioid overdose or poisoning (965.0) and opioid-type drug dependence and abuse (304.0, 304.7, or 305.5) as well as poisoning and adverse effect E-codes (E850.0-E850.2 [accidental poisoning by opioids and related narcotics], E935.0-E935.2 [adverse effects of opioids and related narcotics]). Opioid poisoning or abuse diagnoses primarily reflect misuse (e.g., substance incorrectly administered or given), non-medical use (e.g., without a prescription), and conditions caused by abusive patterns of drug use (e.g., dependence, psychoses), whereas opioid adverse effects (e.g., delirium, tachycardia, vomiting) generally indicate allergic or hypersensitivity reactions and exclude unintentional (accidental) or suicidal overdoses [8]. An opioid intoxication event therefore may involve pharmaceutical (e.g., morphine, codeine, oxycodone, hydrocodone, fentanyl, methadone, buprenorphine) or nonpharmaceutical (e.g., heroin) substances. This case definition enables a more complete representation of opioid-related healthcare utilization, as pharmaceutical opioid consumption and heroin use are increasingly overlapping problems [9, 10]. Opioid-related medical encounters were also examined for alcohol, benzodiazepine (BZD) and non-opioid illicit drug (e.g., cocaine, cannabinoids, hallucinogens and other psychostimulants) involvement, given their well-documented contributions to poisoning fatalities [11–15]. Comorbid conditions, including pain-related diagnoses, major chronic diseases (hypertension, ischaemic heart disease, renal failure/diabetes, cancer, chronic lower respiratory disease [CLRD]), affective disorders, sleep disorders, sexually transmitted infections (STI) and viral hepatitis were assessed from all available diagnosis fields. Additionally, accidental and self-harm or suicidal intents were determined using injury-related E-codes if recorded (E850-E858 [accidental poisoning by drugs] and E950.0-E950.5 [self-inflicted poisoning by drugs]). All other cases, including opioid poisoning or abuse related injuries of other and uncoded intents and excluding opioid adverse effects, were combined into an ‘other opioid misuses’ category in intent-specific analyses.

### Statistical analysis

To determine how opioid intoxications varied across socioeconomic settings, aggregated age and race-specific opioid cases were linked to population characteristics from the US Census five-year (2009–2013) American Community Survey by residential zip code to incorporate median household income and denominator data. To the extent that features of the social environment such as neighborhood income and place of residence influence health outcomes, health services utilization, and the context of drug availability, counts (by age and race) of opioid-related medical encounters within the same zip code may be more similar than those in different zip codes (i.e., counts may be autocorrelated). As such, they were modeled by negative binomial regression with the generalized estimating equations (GEE) to allow for autocorrelation and potential overdispersion (i.e., variance is greater than mean) [16]. The GEE estimation assumed an unstructured working correlation for counts over clusters (i.e., zip codes), and controlled for differences in age, race (restricted to non-Hispanic white, black and Hispanic patients) and male-to-female ratio (per patient’s zip code) as well as unobserved contextual heterogeneities (of the 74 zip codes from the data linkage) in calculating the rate for opioid intoxication and comorbidities by areal-level income. Variations in rates across income quartiles were expressed as multivariable-adjusted rate ratios (using the 4^th^ income quartile as the reference group). The GENMOD procedure of SAS 9.3 (Cary, NC, USA) was used to perform the analysis.

Opioid injury and comorbidities were further examined as a function both of neighborhood income and individual characteristics (age, gender, race, insurance type), with mixed-effects logistic regression to estimate the likelihood for an adverse outcome. Per standard mixed-effects modeling, a random intercept deviation (for each zip code) was adopted to account for the effects of socio-spatial context, that is, interdependencies of opioid-related medical encounters within each zip code. Accidental poisonings were compared with other opioid misuse cases (including suicidal poisonings), suicidal poisonings with other opioid misuse cases (including accidental poisonings), and comorbidity-involved cases with those without the concomitant condition, respectively, to derive multivariable-adjusted odds ratio (AOR) estimates for selected study endpoints across major racial groups (using non-Hispanic white [NHW] as the reference group). The GLIMMIX procedure of SAS 9.3 was used for the mixed-effects analysis.

## Results

For the 3-year study period (2011–2013), there were a total of 4,631 ED visits and 5,016 IHs among residents in Southern Nevada which were attributable to the use of opioids. These ED visits and IHs constituted about a quarter of drug-induced emergency visits and one-half of drug-induced hospitalizations, respectively. The overall rate of opioid-related intoxications was 161.0 per 100,000, with ED visit rates peaking in ages 20–34 (147.7 per 100,000) and IH rates in ages 55 and above (127.8 per 100,000). More than half of opioid intoxication cases were among women, and close to three-quarters among those of non-Hispanic white origin (Table 1). The most common type of insurance among inpatients was private coverage (37.4 %), followed by Medicare (33.1 %) and Medicaid (12.9 %), while self-pay was most common for ED visits (34.3 %), followed by private coverage (28.4 %) and Medicare (14.9 %). More Hispanic patients were treated in ED than in hospitals, and self-pay (as the primary payer) was more likely among Hispanics than other races. Hospitalizations lasted 5–6 days on average, and discharge to home or self-care occurred in almost two-thirds of the patients. Median charges per patient in 2013 were $4,528 (interquartile range: $2,334 to $7,126) in ED visits without hospitalization and $24,659 ($10,604 to $59,622) in IHs. Diagnosis of opioid poisoning or adverse effects was associated with substantial increases in hospital care costs, with median IH charges of $41,329 and $62,757 respectively.

**Table 1:** Opioids-Attributable ED Visits and Inpatient Hospitalizations (IH) by Selected Characteristics, Clark County-NV, 2011-2013

	ED visit		IH		Total Encounters			Alcohol-related			BZD-related			Illicit/recreational drug-related^c^		
	No.	%^a^	No.	%^a^	No.	%^a^	Rate^b^	No.	%^a^	Rate^b^	No.	%^a^	Rate^b^	No.	%^a^	Rate^b^
Total	4,631	100.0	5,016	100.0	9,647	100.0	161.0	697	100.0	11.6	817	100.0	13.6	1,120	100.0	18.7
Age																
< 20 y	408	8.8	283	5.6	691	7.2	43.1	24	3.4	1.5	39	4.8	2.4	122	10.9	7.6
20–34	1,911	41.3	1,450	28.9	3,361	34.8	259.7	204	29.3	15.8	245	30.0	18.9	550	49.1	42.5
35–54	1,478	31.9	1,469	29.3	2,947	30.5	176.1	305	43.8	18.2	320	39.2	19.1	331	29.6	19.8
55+	834	18.0	1,814	36.2	2,648	27.4	186.6	164	23.5	11.6	213	26.1	15.0	117	10.4	8.2
Gender																
Female	2,341	50.6	2,674	53.3	5,015	52.0	168.2	273	39.2	9.2	450	55.1	15.1	483	43.1	16.2
Male	2,290	49.4	2,342	46.7	4,632	48.0	153.9	424	60.8	14.1	367	44.9	12.2	637	56.9	21.2
Race																
Non-Hispanic white	3,342	72.2	3,787	75.5	7,129	73.9	247.1	534	76.6	18.5	616	75.4	21.3	805	71.9	27.9
Non-Hispanic black	455	9.8	458	9.1	913	9.5	135.7	53	7.6	7.9	68	8.3	10.1	119	10.6	17.7
Hispanic	535	11.6	369	7.4	904	9.4	50.7	62	8.9	3.5	78	9.5	4.4	105	9.4	5.9
Other	299	6.5	402	8.0	701	7.3	107.5	48	6.9	7.4	55	6.7	8.4	91	8.1	14.0
Insurance type																
Medicaid	563	12.2	645	12.9	1,208	12.5	--	88	12.6	--	138	16.9	--	168	15.0	--
Medicare	689	14.9	1,661	33.1	2,350	24.4	--	128	18.4	--	190	23.3	--	130	11.6	--
Other public (military, indigent, charity)	336	7.3	366	7.3	702	7.3	--	92	13.2	--	68	8.3	--	159	14.2	--
Private (including workers’ comp)	1,314	28.4	1,878	37.4	3,192	33.1	--	205	29.4	--	222	27.2	--	307	27.4	--
Self-pay	1,590	34.3	413	8.2	2,003	20.8	--	171	24.5	--	185	22.6	--	319	28.5	--
Other/unknown	139	3.0	53	1.1	192	2.0	--	13	1.9	--	14	1.7	--	37	3.3	--
Selected comorbidities^a^																
Chronic bodily pains	696	15.0	2,253	44.9	2,949	30.6	49.2	190	27.3	3.2	295	36.1	4.9	226	20.2	3.8
Affective disorders	885	19.1	1,698	33.9	2,583	26.8	43.1	272	39.0	4.5	425	52.0	7.1	340	30.4	5.7
Hypertension	565	12.2	1,811	36.1	2,376	24.6	39.7	158	22.7	2.6	215	26.3	3.6	158	14.1	2.6
Renal failure/diabetes	244	5.3	1,130	22.5	1,374	14.2	22.9	90	12.9	1.5	149	18.2	2.5	105	9.4	1.8
Chronic lower respiratory disease	255	5.5	892	17.8	1,147	11.9	19.1	83	11.9	1.4	100	12.2	1.7	113	10.1	1.9
Ischemic heart disease	90	1.9	493	9.8	583	6.0	9.7	30	4.3	0.5	55	6.7	0.9	44	3.9	0.7
Sleep disorders	57	1.2	341	6.8	398	4.1	6.6	26	3.7	0.4	37	4.5	0.6	34	3.0	0.6
STI/viral hepatitis	68	1.5	265	5.3	333	3.5	5.6	56	8.0	0.9	38	4.7	0.6	64	5.7	1.1

^a^Comorbid conditions are not mutually exclusive. Crude rates are based on Census total population estimates (average 2011–2013)

^b^Rates are per 100,000 Census population estimates by demographic subgroups unless specified otherwise

^c^Illicit/recreational drugs include cocaine, cannabinoids, hallucinogens and other stimulants with abuse potential

--Not estimated

Of the 9,647 opioid use or misuse related medical encounters in 2011–2013, 23.9 % (2,305) were coded as unintentional injury and 9.6 % (930) suicidal, whereas adverse effects were the first-listed diagnosis in about 17 % (1,663) of cases. (Of the opioid poisoning cases assigned an injury-related E-code [3,547], 65 % were classified as accidental and 26.2 % suicidal.) Females aged 55 years and above comprised almost 40 % (639) of all opioid adverse effect cases, versus 16.6 % (589) of opioid poisoning cases. Fatalities (49 inpatient and 4 ED cases at time of discharge) occurred predominantly among inpatients (at a rate of 1 %), to those aged 55 and above, and were most frequently related to accidental exposure to opioid drugs (22 accidental vs. 13 suicidal poisonings). In comparison to patients hospitalized for non-opioid related drug intoxications, opioid cases were more likely to be women, aged 55 years and above, to have private coverage or Medicare as the primary payer, to have longer hospital stays, and to be transferred to another facility or self-care.

As well, females made up a larger proportion of suicidal (60.9 %) than accidental poisonings (50.4 %), and ages 35–54 the largest proportion of suicidal poisonings (38.8 %), whereas ages 55 and above the largest proportion of accidental poisonings (36 %). Compared with opioid overdose deaths identified from vital records (data not shown), opioid-related medical encounters had lower involvement of alcohol (7.2 %), BZD (8.5 %), or illicit/recreational drugs (11.6 %) (diagnoses for substances involved are not mutually exclusive). Nonetheless, rates of alcohol (15.7 %) or BZD (29.5 %) involvement were higher in suicidal than accidental poisonings (8.5 % and 18.3 % respectively). Alcohol co-use among opioid cases was most commonly found in males aged 35–54, BZD co-use in similarly-aged females, and illicit drug co-use in males under 35 years of age (Table 1).

The most frequent comorbidities among opioid-related ED or hospital visits were chronic bodily/musculoskeletal pains and affective disorders (e.g., depression, bipolar disorders, depressive/affective psychoses), listed as comorbid diagnoses in 30.6 and 26.8 % of the examined opioid cases respectively (comorbid conditions are not mutually exclusive) (Table 1). Chronic bodily pains were more pronounced among IHs (44.9 %) than ED visits (15 %), and affective disorders (19.1 %) more notable than chronic bodily pains among ED visits. Other conditions commonly found among opioid cases were hypertension (24.6 %), renal failure/diabetes (14.2 %), CLRD (11.9 %), ischaemic heart disease (6 %), sleep disorders (4.1 %), and STI/viral hepatitis (3.5 %). ED service and IH costs increased substantially with a major chronic disease (hypertension, ischaemic heart disease, renal failure/diabetes, cancer, CLRD) comorbidity (from $4,299 and $14,659 to $6,284 and $43,686 in median ED and IH charges in 2013, respectively).

Of the 3,923 opioid-related medical encounters with a pain co-diagnosis, 75.2 % (2,949) were associated with chronic bodily pains, 16.1 % (633) with a psychological disorder or psychogenic pain, 13.1 % (513) with a non-poisoning injury or acute pain, 8.5 % (334) with neuropathy, 7.4 % (292) with headache, and 5.6 % (219) with cancer (pain-related diagnoses are not mutually exclusive). Whereas 41 % of all opioid cases had one or more pain-caused conditions, the proportion rose to 64 % (1,068) among cases diagnosed as adverse effects. As well, pain care was bound up with the management of other physical ailments, given the high co-occurrence of pain (2,244 or 65 %) among those with a major chronic disease diagnosis (3,453).

**Fig. 1:**
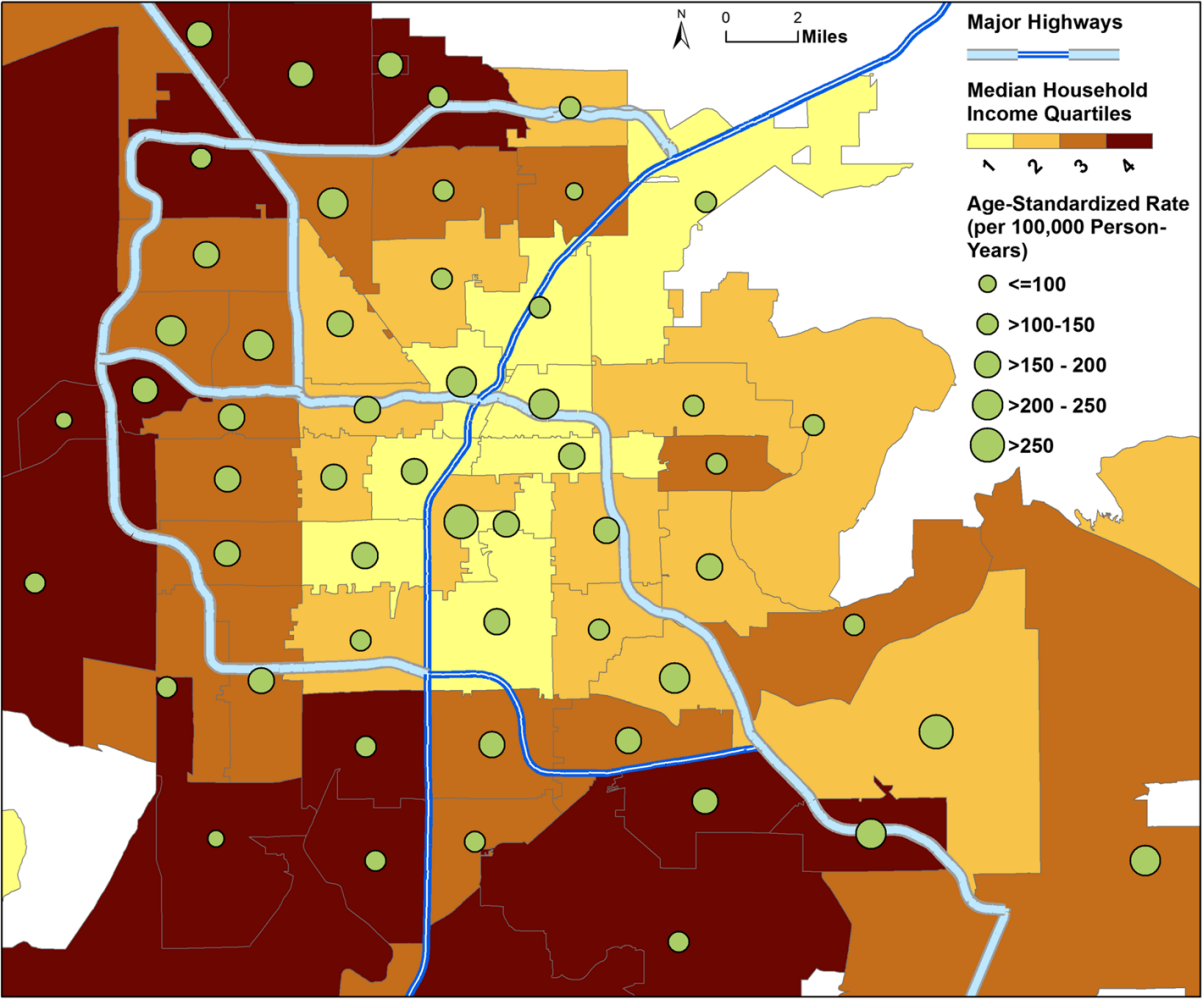
Rate of Opioid-Involved Medical Encounters by Residential Zip Code, Southern Nevada Metro Enlargement, 2011-2013

**Fig. 2:**
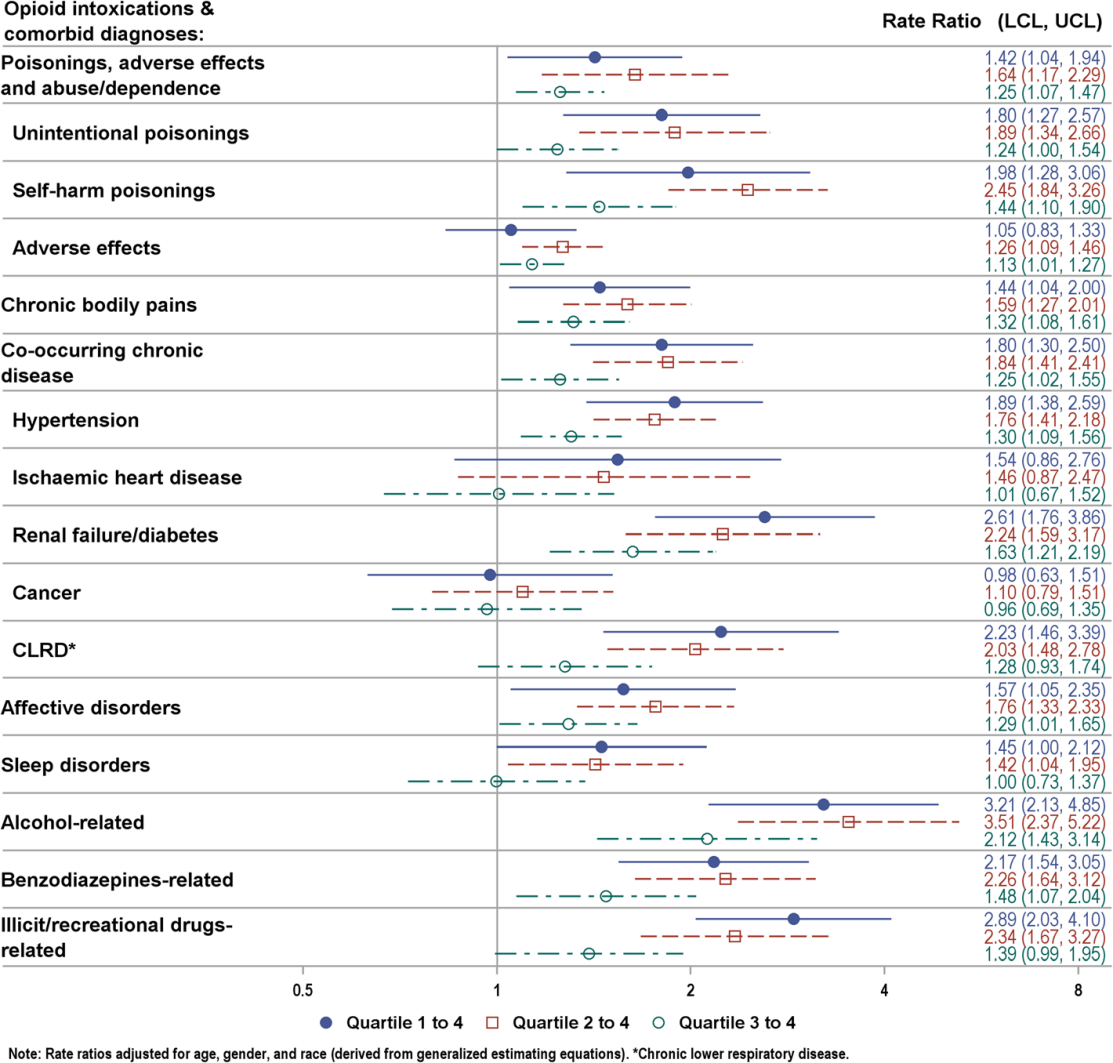
Income Inter-Quartile Rate Ratios with 95 % Confidence Limits for Opioid-Involved Medical Encounters, Clark County-Nevada, 2011-2013

Opioid intoxications were more common in low-income communities (Fig. 1; zip code used as the enumeration area), and this socioeconomic disparity persisted after accounting for the effects of age, gender and race. Figure 2 shows the multivariable-adjusted rate ratios for opioid intoxication and selected comorbid diagnoses, comparing zip codes grouped into lower income quartiles (on the basis of median household income) to the richest quartile (quartile 4). There was a general pattern of increase in opioid intoxications and examined comorbidities for individuals living in low-income areas. The multivariable-adjusted rate for opioid-related healthcare utilization was 42 % higher in the poorest (median income $33,029) vs. richest ($76,030) quartile during the study period, whereas for opioid cases coded as either accidental or suicidal poisoning, related medical encounters showed greater inter-quartile disparities, with rates about twice as high in the poorest vs. richest quartile. Marked socioeconomic differentials were also apparent in the prevalence of chronic bodily pains, chronic diseases (except for cancer), and affective disorders among opioid cases, with residents of poorer quartiles at much higher risk. The inter-quartile (quartile 1 vs. 4) rate increases for comorbidities such as chronic bodily pains (44 %), hypertension (89 %), renal failure/diabetes (2.6 times), CLRD (2.2 times), and affective disorders (57 %) were all statistically significant. Similarly, poorer neighborhoods retained significantly higher rates for non-opioid substance co-use: the poorest quartile had about 3 times the rate for alcohol or illicit drug co-use, and twice that for BZD co-use compared with the richest quartile.

**Fig. 3:**
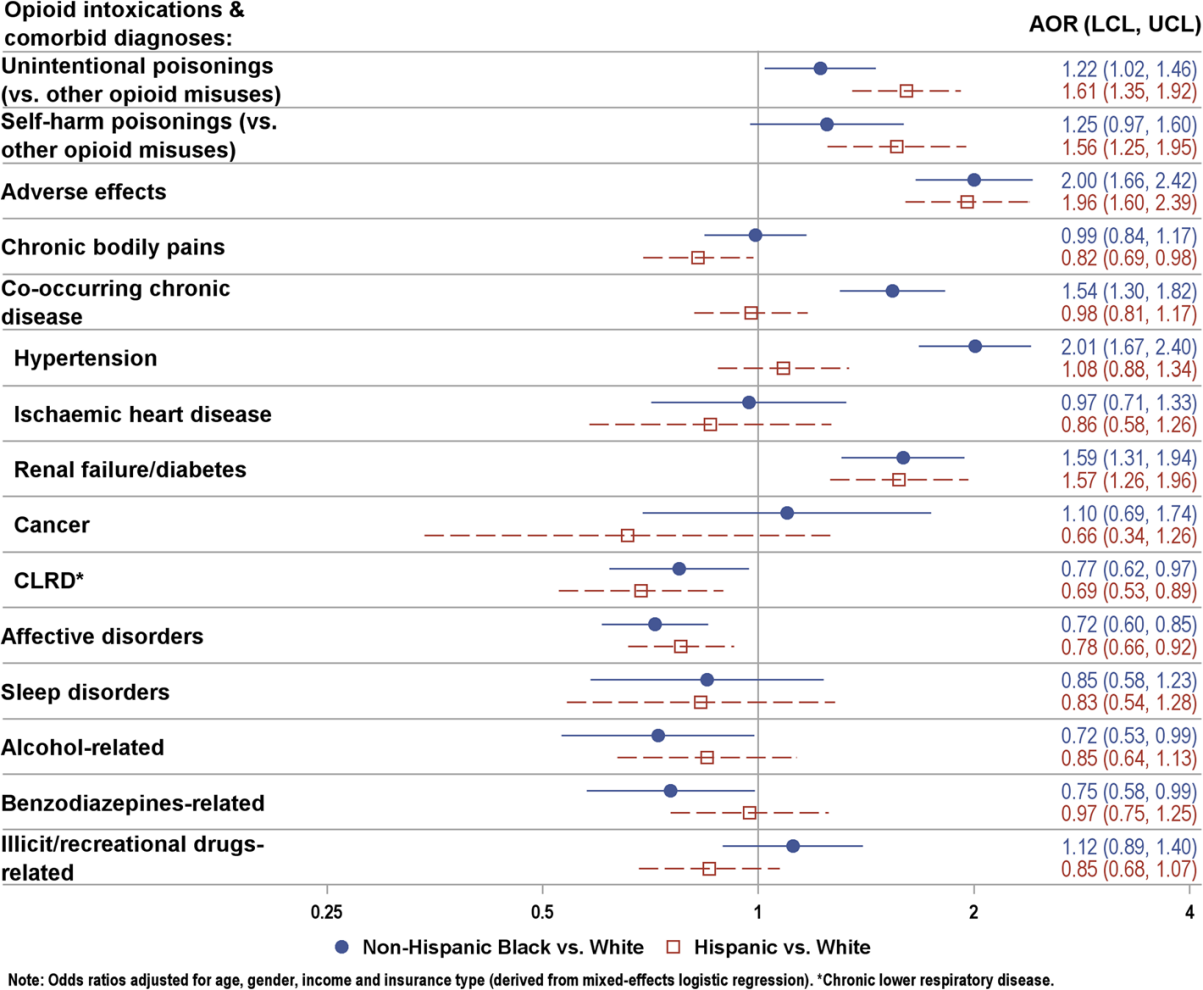
Odds Ratios with 95 % Confidence Limits for Opioid-Involved Medical Encounters across Select Racial Groups, Clark County-Nevada, 2011-2013

In addition, opioid morbidity and comorbidity patterns varied by race after adjustment for age, gender, insurance type, and neighborhood income (Fig. 3). Among opioid cases treated in ED or hospital and relative to NHWs, the multivariable-adjusted odds of accidental opioid poisoning (vs. other opioid misuse related intoxications) were 22 % higher for non-Hispanic blacks (NHB), and 61 % higher for Hispanics. Similar disparities were seen for suicidal opioid poisoning (vs. other opioid misuse related intoxications), with the odds 56 % higher among Hispanics and elevated albeit nonsignificantly among NHBs. Also, NHBs and Hispanics had substantially increased odds of opioid adverse effects (vs. opioid misuse related intoxications), which were twice the odds for NHWs. Further, chronic disease comorbidity was greater among NHBs, due in large part to greater odds of co-occurring hypertension (AOR of 2.01) and renal failure/diabetes (1.59) in NHBs. As well, Hispanics had significantly greater odds for concomitant renal failure/diabetes (1.57), albeit comparatively lower odds for other chronic disease comorbidities than NHWs. On the other hand, opioid drug abuse or dependence related disorders, alcohol or BZD co-use, and correlates of opioid intoxication such as chronic bodily pains, affective disorders and sleep disorders were more prevalent among NHWs than nonwhites.

## Discussion

Using de-identified ED and hospital discharge records, we evaluated health service utilization associated with opioid intoxication and its relationship with sociodemographic and clinical factors in a large metropolitan area (the Las Vegas–Paradise MSA). We observed substantial opioid morbidity burden, as measured by both healthcare utilization and expenditure. Opioid use and misuse were implicated in over 1,500 emergency visits and 1,700 IHs annually during the study period, and opioid-related intoxications cost Southern Nevada’s health system about $9.3 million in ED service costs and $92.5 million in inpatient healthcare costs in 2013. Important opioid comorbidities include pain, affective disorders, and chronic physical ailments, with a high co-occurrence of pain among patients affected by a major chronic condition. These findings suggest that enhanced overdose screening (e.g., urine toxicology, ascertainment of substance use history), especially of patients being treated for pain, is warranted as a key component of prevention-oriented care.

Indeed, pain management is a significant area of healthcare expenditure, amounting to two-thirds ($67 million) of the total ED and IH charges in Southern Nevada for opioid-related intoxications in 2013. Further, the significance of pain and coexistent emotional or physical problems extend beyond opioid overdose or addiction and related healthcare costs. It was estimated that pain-attributable medical care contributed about 47 % of the annual economic costs of pain in the United States, while pain-induced workplace costs (e.g., lost productivity) made up the rest of the related economic burden [17]. Importantly, cost estimates are necessarily conservative as the effects of pain on social functioning, emotional well-being and quality of life are difficult if not impossible to monetize. As such, there are compelling economic and moral imperatives to make pain prevention a public health priority [18].

The current analyses indicate that there are leverage points for the mitigation of pain and opioid-related healthcare costs. Most notably, opioid-related disorders are highly comorbid, and low-income neighborhoods experience a disproportionate burden of opioid intoxications and comorbidites ranging from prevalent chronic diseases to affective disorders. As low-income patients were more likely to use acute hospital care and less likely to access primary care or obtain specialty referral than middle- and high-income patients [19–22], a case could therefore be made for improving the capacity of and access to evidence-informed pain care and behavioral health services in primary care settings on the grounds of the possible improvements in health system efficiency that could result. This includes developing comprehensive screening and assessment tools to enhance the critical detection and referral roles of primary care services, implementing quality assurance standards to encourage multi-disciplinary pain management approaches (e.g., medical and psychosocial interventions), aligning insurance incentives with standards of care, building the competencies of the substance abuse and mental health workforce, and focusing prevention and outreach services on low-income populations.

Results from this study indicate that NHWs had greater opioid-related health service utilization than nonwhites. In light of previous evidence on similar racial disparities [23–25], this pattern may suggest that nonwhite individuals did not experience the pain reduction benefits or associated risks of opioid medications to the same degree as NHWs. This racial disparity may relate to sociocultural differences in pain perception and reporting, patient-provider interactions, potential barriers to analgesic treatment, and differential opioid-taking behavior [23, 26–29]. As such, future work to explore patient (e.g., treatment preferences, presentation, copying strategies) and provider (e.g., treatment modalities, prescribing patterns) related factors as well as information derived from patient-provider interactions could contribute to a better understanding of the racial disparities in healthcare utilization. As well, additional studies on the nature and extent of pain disparities in diverse populations could help clarify when higher medical encounter rates are the result of avoidable health service utilization and when they reflect a greater need for pain treatment and management.

While administrative data are unable to address pain disparities or clinical practice variations in pain care, the higher rates of opioid abuse or dependence related disorders and additional psychotherapeutics (e.g., BZD) use among NHWs emphasize the need for vigilance about possible progression from legitimate medical use to substance abuse and dependence in this population. The finding of elevated odds for affective disorders among NHWs compared with nonwhites also deserves mention, and supports previous reports of higher psychiatric comorbidity in vulnerable populations [3, 5, 30, 31]. Clearly, the association of chronic pain and psychiatric disorders, with their underlying risk and protective factors, should be a focus in etiologic and prevention research. On the other hand, the relative risks for opioid poisoning (vs. abuse or dependence) and opioid adverse effects (vs. poisoning and abuse/dependence) were significantly higher among NHBs and Hispanics than among NHWs, suggesting a need for targeted overdose education, suicide screening, and close monitoring of complications among minority patients. Further, opioid poisoning and adverse effects were associated with higher healthcare costs and poorer clinical status, as reflected in their higher-weighted case-mix evaluations (data not shown) when compared with abuse or dependence diagnoses. This suggests that broader prevention initiatives—such as preventive care and early disease management—may go some way towards mitigating the risks of opioid comorbidities and related healthcare costs.

The current study expands the scope of existing literature on opioid-related morbidity by incorporating the spectrum of opioid injury comprising poisoning, abuse or dependence, and adverse effects. Most prior studies have focused on fatal/nonfatal overdoses (exclusive of adverse effects) or non-medical use. Therefore, very little information is available on potential factors associated with both opioid misuse and adverse effect events. We characterized medical and non-medical opioid users’ patterns of healthcare utilization by estimating their association with sociodemographic and clinical risk factors in large-scale, population-based samples. Our findings further describe the relationship of opioid intoxication risk to socioeconomic status and the differential clinical characteristics (such as comorbid diagnoses) among demographic subgroups. We noted elevated risk for opioid overdose and adverse effects among nonwhite populations, and among NHW populations, increased risk for opioid abuse and psychiatric problems. These data underscore the importance of a multifaceted prevention approach that encompasses both mental health strategies and clinical interventions (such as heightened surveillance of at-risk patients who may benefit from other treatment modalities).

There are a number of limitations to this study. First, its cross-sectional nature precludes the demonstration of a cause-and-effect relationship between healthcare utilization and individual or ecologic covariates. Second, lack of adjustment for potential confounders including individual (e.g., disability, medical history, lifestyle behaviors) and socioeconomic (e.g., employment, education, neighborhood resources) correlates could have resulted in overestimating the magnitude of intoxication risks due to measured covariates. In addition, the use of ecologic covariate such as zip code level male-to-female ratio in income-attributable effect estimation may have inadequately adjusted for the possibility of confounding by gender. Third, the ICD-9-CM diagnosis codes for opioid-type drug abuse/dependence do not differentiate between prescription and illicit (e.g., heroin) opioid-involved disorders. Accordingly, underestimation of illicit drug-related intoxications likely led to a conservative bias with fewer differences observed between sociodemographic groups. Fourth, we were unable to account for repeat medical encounters as deidentified data sources preclude follow-up of patients over time. Last, data available for this study do not permit in-depth examination of factors contributing to the opioid overdose risk that occurred disproportionally more often among residents in Southern Nevada than in the rest of the nation. Future work to understand the geographic patterns of opioid intoxication could therefore facilitate identification of economic and socio-environmental exposure pathways that would inform operationally specific policy interventions. Whereas our findings reinforce income-mediated behavioral and mental health disparities, other research has indicated that higher levels of social spending for low-income residents significantly reduced health and life expectancy disparities along the income gradients [32]. Thus, it is possible that resource commitment to the mitigation of income inequality and social disinvestment is a policy lever with importance to overdose prevention.

## Conclusions

This study extends previous research by evaluating health service utilization associated with opioid intoxication and its relationship with sociodemographic and clinical factors in an urban setting. There were consistent patterns of disparities in healthcare utilization across sociodemographic groups for opioid-associated disorders, with low-income neighborhoods experiencing a disproportionate burden. Further initiatives to evaluate the determinants of overdose and abuse and to implement targeted response efforts are needed. At the population and health system level, intervention strategies informed by the etiology of pain including its physical and psychosocial correlates would prove beneficial in reducing opioid-associated healthcare utilization.

## Abbreviations

AOR: Multivariable-adjusted odds ratio
BZD: Benzodiazepine
CLRD: Chronic lower respiratory disease
ED: Emergency department
GEE: Generalized estimating equations
IH: Inpatient hospitalizations
LCL: Lower confidence limit
MSA: Metropolitan statistical area
NHB: Non-Hispanic black
NHW: Non-Hispanic white
STI: Sexually transmitted infections
UCL: Upper confidence limit
US: United States
